# Differential regulation of undecylprodigiosin biosynthesis in the yeast-scavenging *Streptomyces* strain MBK6

**DOI:** 10.1093/femsle/fnab044

**Published:** 2021-04-21

**Authors:** Baral Bikash, Siitonen Vilja, Laughlin Mitchell, Yamada Keith, Ilomäki Mikael, Metsä-Ketelä Mikko, Niemi Jarmo

**Affiliations:** Department of Biotechnology, University of Turku, FIN-20014 Turku, Finland; Department of Biotechnology, University of Turku, FIN-20014 Turku, Finland; Department of Biotechnology, University of Turku, FIN-20014 Turku, Finland; Department of Biotechnology, University of Turku, FIN-20014 Turku, Finland; Department of Biotechnology, University of Turku, FIN-20014 Turku, Finland; Department of Biotechnology, University of Turku, FIN-20014 Turku, Finland; Department of Biotechnology, University of Turku, FIN-20014 Turku, Finland

**Keywords:** DNA-binding proteins, prodigiosin, antibiotic biosynthesis, *Streptomyces*, transcription factors

## Abstract

*Streptomyces* are efficient chemists with a capacity to generate diverse and potent chemical scaffolds. The secondary metabolism of these soil-dwelling prokaryotes is stimulated upon interaction with other microbes in their complex ecosystem. We observed such an interaction when a *Streptomyces* isolate was cultivated in a media supplemented with dead yeast cells. Whole-genome analysis revealed that *Streptomyces* sp. MBK6 harbors the *red* cluster that is cryptic under normal environmental conditions. An interactive culture of MBK6 with dead yeast triggered the production of the red pigments metacycloprodigiosin and undecylprodigiosin. *Streptomyces* sp. MBK6 scavenges dead-yeast cells and preferentially grows in aggregates of sequestered yeasts within its mycelial network. We identified that the activation depends on the cluster-situated regulator, *mbkZ*, which may act as a cross-regulator. Cloning of this master regulator *mbkZ* in *S. coelicolor* with a constitutive promoter and promoter-deprived conditions generated different production levels of the red pigments. These surprising results were further validated by DNA–protein binding assays. The presence of the *red* cluster in *Streptomyces* sp. MBK6 provides a vivid example of horizontal gene transfer of an entire metabolic pathway followed by differential adaptation to a new environment through mutations in the receiver domain of the key regulatory protein MbkZ.

## Teaser

Genome analysis of a prodigiosin producing Streptomyces isolate revealed differences in key regulatory genes, probably connected with the responsiveness of this strain to yeast.

## INTRODUCTION

Soil microbiota represents one of the most complex and diverse ecosystems found on our planet (Crowther *et al*. 2019). This nutrient scarce habitat hosts interacting communities of microorganisms, especially bacteria and fungi, competing for resources. A particularly interesting facet of these communities is the ability of the microbes to communicate with each other using secondary metabolites. Examples include initiation of an exploratory growth phase in generally stationary *Streptomyces* bacteria upon encountering yeast (Jones *et al*. 2017) and the chemical warfare between *Streptomyces* and *Aspergillus* where the microbes respond to chemical stimuli from each partner (Nützmann *et al*. 2011; Khalil *et al*. 2018).

Actinobacteria are prolific producers of biologically active natural products, which have contributed approximately two thirds of antibiotics and one third of anticancer agents in clinical use (Newman and Cragg 2020). Genome sequencing projects have revealed that *Streptomyces* typically harbor few tens of biosynthetic gene clusters (BGC) that encode chemically diverse secondary metabolites (Bentley *et al*. 2002). However, many of these BGCs remain dormant under laboratory monocultures and require environmental signals from the soil microbiota community for activation of gene transcription. Numerous biotechnological applications have been developed in order to activate microbial secondary metabolic pathways for production of novel bioactive compounds (Baral, Akhgari and Metsä-Ketelä 2018).

Undecylprodigiosin is a well-studied bioactive substance (Tsao *et al*. 1985; Malpartida *et al*. 1990) produced, among others, by *Streptomyces coelicolor* (Cerdeño, Bibb and Challis 2001), which possesses antitumor, immunosuppressant, antifungal and antimalarial activities (Stankovic *et al*. 2014). Recently, the production of prodigiosins has been implicated as an agent of programmed cell death in the life cycle of *S. coelicolor* (Tenconi *et al*. 2018), but their production has also been reported to increase when in contact with *Bacillus subtilis* (Luti and Mavituna 2011).

We have previously noted that *Streptomyces* may respond to the presence of yeast by triggering production of extracellular cholesterol oxidase (Yamada *et al*. 2019). This study continues the characterization of *Streptomyces*–yeast interactions. The strain *Streptomyces* sp. MBK6 isolated from a soil sample was found to produce a red substance in the presence of yeast. The products were identified as undecylprodigiosin and metacycloprodigiosin and the corresponding BGC denoted *mbk* was readily observed in the genome sequence of *Streptomyces* sp. MBK6. Comparison with the *S. coelicolor red* (undecylprodigiosin) BGC (Cerdeño, Bibb and Challis 2001) indicated high similarity, however dissimilarity was found in the area encoding the primary regulatory gene *redZ/mbkZ*. We therefore tested the effect of *mbkZ* on prodigiosin production in the presence and absence of yeast in *Streptomyces* sp. MBK6 and *S. coelicolor*, and on binding of MbkZ to promoters of the BGC-specific regulatory genes.

## MATERIALS AND METHODS

### Biological materials

The yeast interaction media were Y2 (20 g/L glycerol, 2.5 g/L autoclaved bakery yeast, 2.5 g/L yeast extract, 1 g/L K_2_HPO_4_, 1 mL/L trace salts solution [FeSO_4_·7H_2_O 1 g/L, MnCl_2_·4H_2_O 1 g/L, ZnSO_4_·7H_2_O 1 g/L]) and YE (the same without yeast). *S. coelicolor* M145, *S. lividans* TK24 and *Streptomyces* sp. MBK6 were transformed by protoplast transformation (Kieser *et al*. 2000). Plasmid constructions, primers and synthetic genes used are shown in the supplementary materials.

### Isolation of Streptomyces sp. MBK6

Soil samples originating from south-western Finland were collected and bacteria with *Streptomyces*-like colony morphology were isolated by serial dilution and tested for antibiotic activity against *Kocuria rhizophila* ATCC 9341 (‘*Micrococcus luteus*’ (Tang and Gillevet 2003)) by an agar plug – zone of inhibition test as described (Barnard 1994). *Streptomyces* sp. MBK6 was noted due to its strong red color.

### Isolation and purification of the red pigments

A 3 l fermentation was inoculated with 50 mL of *Streptomyces* sp. MBK6 preculture in 26 g/L whole yeast, 9 g/L glucose, 2 g/L CaCO_3_, 2 g/L NH_4_NO_3_ in tap water. The fermentation continued for 5 days at 30°C with stirring and aeration. Cells were collected by centrifugation for 20 min at 7025 × *g*. The red compounds were extracted from centrifuged cell pellets with 1:1:1 methanol: toluene: 0.1 M phosphate buffer pH 7. The toluene phase was subsequently washed with phosphate buffer. The samples were dried and dissolved in a minimal volume of 9:1 chloroform: methanol before applying to a normal-phase silica column and a gradient from 10 to 100% methanol in chloroform was run. Fractions of interest were further purified by preparative HPLC (LC‐20AP, model; CBM‐20A, Shimadzu, SunFire Prep C18, 5 μm 10 × 250 mm, Waters) using a gradient from 15% methanol with 0.1 formic acid to 100% methanol.

### Analyses of compounds

The compounds were analysed by HPLC (SCL-10Avp HPLC with an SPD-M10Avp diode array detector, Shimadzu, Tokyo, Japan) using 15% MeOH with or without 0.1% formic acid to 100% MeOH using a KINETEX column (2.6u C18 100Å; 100 × 4.6 mm, Phenomenex, Torrance, CA, USA). Purified compounds were analysed by high resolution mass (MicrOTOF-Q, Bruker Daltonics, Bremen, Germany) by direct injection in positive ionization mode. For NMR analysis the purified and desiccated compounds were dissolved in MeOD or CDCl3. 1D measurements: ^1^H and ^13^C NMR and 2D measurements (COSY, heteronuclear multiple bond correlation, HMBC, heteronuclear single quantum coherence HSQCDE, heteronuclear single quantum correlation, edited) were performed with following instruments: 600 MHz Bruker AVANCE-III NMR-system with a liquid nitrogen cooled Prodigy TCI (inverted CryoProbe) and a 500 MHz Bruker AVANCE-III NMR-system with a liquid nitrogen cooled Prodigy BBO (CryoProbe). The signals were internally referenced to tetramethylsilane. Topspin (Bruker Biospin) was used for spectral analysis.

### Genome sequencing

*Streptomyces* sp. MBK6 was cultured in 30 mL of GYM media with 0.5% glycine at 30°C for 2 days shaking at 300 rpm and then pelleted. Genomic DNA was extracted (Nikodinovic, Barrow and Chuck 2003 with slight modifications). Quality control and the PCR-free shotgun library (Illumina, San Diego, CA, USA) was prepared at the Finnish Functional Genomics Centre (Turku, Finland). A single lane of an Illumina MiSeq v3 sequencer was used to produce 2 × 300 bp reads. The quality of the reads was manually checked before and after error correction using FASTQC (v0.11.2; Andrews 2010). The reads were assembled using A5-miseq (v20150522; Coil, Jospin and Darling 2015), contiguated with ABACAS (v1.3.1; Assefa *et al*. 2009) using *Streptomyces albus* NK660 (CP007574.1) as the reference, and the gaps were filled using IMAGE (v2.4.1; Tsai, Otto and Berriman 2010). The final assembly was annotated using RAST (Brettin *et al*. 2015) and evaluated for completeness using BUSCO (v1.22; Simão *et al*. 2015). All programs were used with the default parameters and run on the CSC—IT Center for Science's Taito super-cluster (Espoo, Finland).

The sequencing of *Streptomyces* sp. MBK6 resulted in 4 530 672 reads that were error corrected and trimmed down to 4 379 905 reads, which were then *de novo* assembled into 65 contigs. ABACAS ordered and aligned the contigs into 25 scaffolds with an N50 of 664 525 bp. The final genome assembly is 7.6 Mbp with a GC content of 72.7% and median coverage of 144x. The BUSCO analysis searched for 40 single-copy orthologs and found 40 (100%) were complete. Out of the 40 complete BUSCOs, two were found multiple times throughout the assembly. Furthermore, no BUSCO was identified as fragmented. The genome was deposited in DDBJ/ENA/GenBank under the accession number JACERG000000000. The version described in this paper is version JACERG010000000. The biosynthetic gene cluster was deposited in MIBiG under the accession number BGC0002090​.

### Expression of mbkZ

The gene of size 1045 bp (Supplementary methods) was purchased from Genewiz (South Plainfield, NJ 07080, USA) and separately subcloned as a *Bam*HI-*Hin*dIII fragment in pIJE486 and pIJ486 to create pIJE486-MbkZsyn (*ermEp-mbkZp*) and pIJ486-MbkZsyn (*mbkZp*) plasmids, respectively. To delete the native promoter (pIJE486-MbkZnop (*ermEp*)) the *Kpn*I-*Hin*dIII fragment was first subcloned in pBADHisBΔ (Kallio *et al*. 2006), and subsequently subcloned as a *Bam*HI-*Hin*dIII fragment in pIJE486. These plasmids were then transformed into *S. coelicolor* M145 and *S. lividans* TK24 through protoplast transformation (Kieser *et al*. 2000). The plasmids were also transformed into *Streptomyces* sp. MBK6 by the protoplast technique, but only for the pIJE486-MbkZsyn (*ermEp-mbkZp*) construct was the transformation successful.

### Assay of total prodigiosins in cultures

Cells from 1 mL of culture were pelleted at 20 000 × *g* for 5 min, and the pellet extracted for 4 h with a mixture of methanol and toluene (400 µL each) in a rotary mixer. 400 µL of 1 mol/L NaOH was added, and after mixing and centrifugation (20 000 × *g*, 1 min) the toluene layer was transferred to a new tube. 400 µL of 1 mol/L HCl was added, mixed and centrifuged as before, and the toluene layer was separated. After diluting to 1 mL, prodigiosin concentration was assessed as absorption at 538 nm (Multiskan Go, Thermo Scientific, Waltham, MA, USA). The methodology was adapted from Kim *et al*. (2007).

### Protein expression and purification

*Escherichia coli* TOP10 cells transformed with pBADHisBΔ-MbkZ were used for protein expression. Precultures were grown overnight in 40 ml of Luria–Bertani (LB) medium with 100 µg/mL ampicillin in 250 mL Erlenmeyer flasks at 30°C and 250 rpm. 400 ml cultures of LB-medium were inoculated with 4 mL of preculture and grown at 30°C and 250 rpm to an OD_600_ of 0.6, whereupon protein production was induced with addition of 0.02% (w/v) l-arabinose and the cultures were left to grow overnight at room temperature and shaking at 180 rpm. Cells were collected through centrifugation at 12 000 × *g* for 20 min at 4°C, resuspended in 20 mL wash buffer (5 mM imidazole, 10% glycerol, 50 mM Tris, 300 mM NaCl and pH 7.5) and disrupted by sonication (Soniprep 150, MSE). 1% Triton X-100 was added, and samples were centrifuged for 50 min at 43 500 × *g* and 4°C to pellet cell debris. The supernatant containing His-tagged MbkZ was mixed with TALON Superflow affinity resin (Clontech, Mountain View, CA, USA) and left to rotate at 4°C for 60 min. The resin was washed with wash buffer and protein was eluted with 2.5 mL elution buffer (250 mM imidazole, 10% glycerol, 50 mM Tris, 300 mM NaCl and pH 7.5). The samples were transferred to a storage buffer (10% glycerol, 100mM Tris, 600 mM NaCl and pH 7.5) using a PD-10 (GE Healthcare, Chicago, IL, USA) column following standard protocol by the manufacturer. Glycerol was added to a total concentration of 40% and the protein was stored at −20°C. The size and purity of the protein was determined by SDS-PAGE and the concentration was measured using the Bradford method (Bradford 1976).

### Electrophoretic mobility shift assay (EMSA)

Genomic DNA of *Streptomyces* sp. MBK6 and *S. coelicolor* was extracted following Nikodinovic, Barrow and Chuck (2003), with the exception that no achromopeptidase was added. DNA fragments of the *mbkD* (453 bp) and *redD* (423 bp) promoter regions were amplified through PCR from the genomic DNA of *Streptomyces* sp. MBK6 and *S. coelicolor*, respectively. A fragment from within the protein coding region of *mbkX* was also amplified and used as a negative control. One primer of each pair (Supplementary methods) was labelled with the fluorescent tag DY682, to incorporate a 5’-label in all DNA fragments. Binding reactions of labelled DNA and MbkZ were done in 20 µL, with binding conditions of 20 mM HEPES, 50 mM (NH_4_)_2_SO_4_, 1 mM DTT, 50 mM KCl, 5 mM MgCl_2_, 1% (v/v) Tween 20, 13.3 mM Tris, 80 mM NaCl, 8% (v/v) glycerol, 0.63 nM DNA and 266 nM protein. After a 30-minute incubation at room temperature, 2 µL of a loading buffer containing 0.25x TBE-buffer (22.25 mM Tris Base, 22.25 mM Boric acid and 0.5 mM EDTA) and 60% glycerol was added, whereafter 15 µL of the reaction mixture was loaded on a pre-cooled 2% TBE agarose gel and run at 4°C for 1 h in pre-cooled 1x TBE. The fluorescence of the DNA was visualized with a Li-Cor Odyssey 9120 imaging system (LI-COR).

## RESULTS

### Isolation and growth of *Streptomyces* sp. MBK6

**Figure 1.**
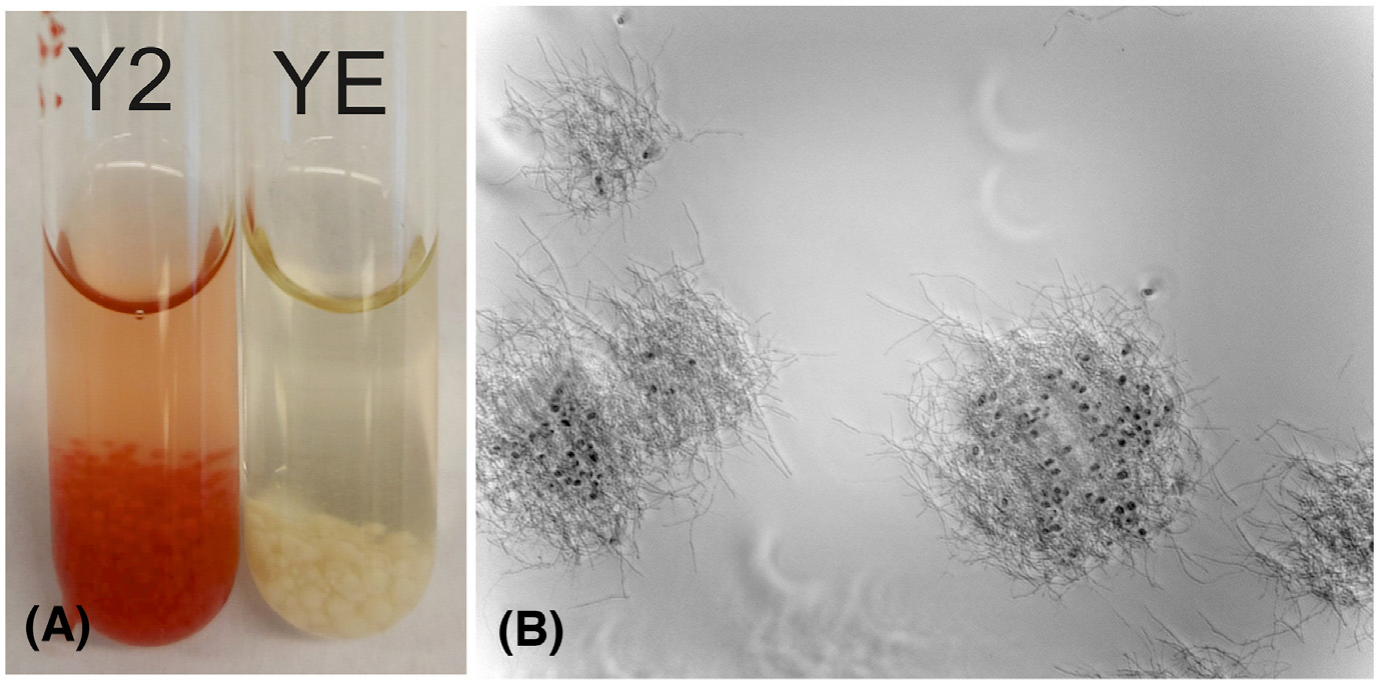
**(A)** Parallel cultures of MBK6 in Y2 (with yeast) and YE medium (without yeast) after 4 days of culture, demonstrating stimulation of red prodigiosin production. **(B)** Sample of Y2 medium, inoculated with a spore suspension of MBK6, after 3 days of culture. Phase contrast microscopy, 100x magnification. The spherical objects are yeast cells; nearly all of them have been sequestered by the growing mycelium.

*Streptomyces* sp. MBK6 was isolated from a soil sample from south-western Finland and produced a bright red antibiotically active compound on plates and in liquid culture when grown in a medium containing whole autoclaved yeast cells. The initial aim was to find new *Streptomyces* strains that would be stimulated by yeast, like the cholesterol oxidase-producing *Streptomyces lavendulae* YAKB-15 (Yamada *et al*. 2019). Under co-culture conditions with yeast, the strain grows as a highly dispersed culture, the mycelium adheres to the yeast cells and the yeast is sequestered in the growing mycelial clumps (Fig. 1). In the presence of yeast, *Streptomyces* sp. MBK6 produces red pigments early, typically on the second day of culture.

### Identification of the pigment as a mixture of undecylprodigiosin and metacycloprodigiosin

*Streptomyces* sp. MBK6 and yeast were co-cultivated in large scale and two red compounds were purified using multiple two-phase organic extractions and chromatography techniques to produce samples suitable for analysis by NMR. Both compounds were analysed by 1D (^1^H and ^13^C) and 2D measurements by NMR and by HR-MS.

The compounds were identified as metacycloprodigiosin (1) (Wasserman, Rodgers and Keith 1969) and undecylprodigiosin (2) (Tsao *et al*. 1985; Fig. 2). Detailed structural analysis can be found in Supporting information (Table S1 and Figures S1–S12, Supporting Information).

**Figure 2.**
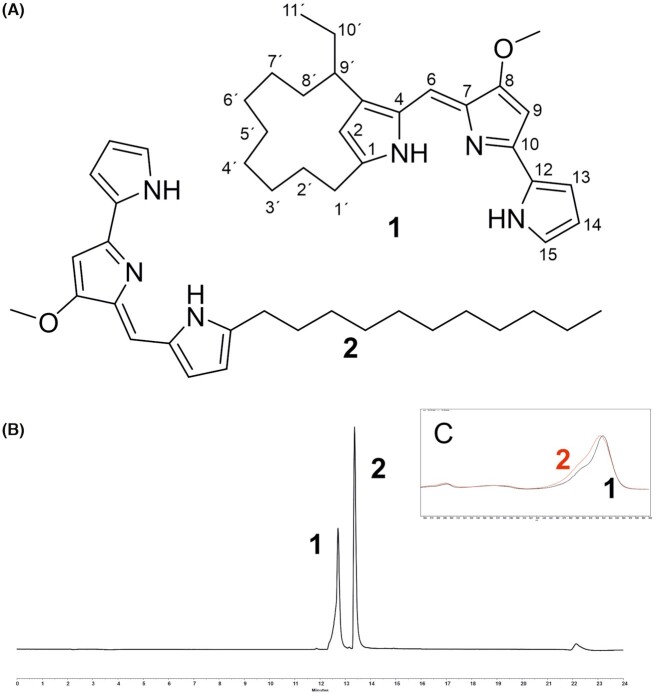
Compounds related to the study. **(A)** metacycloprodigiosin (1) and undecylprodigiosin (2). **(B)** HPLC chromatogram of MBK6 culture extract shown at 530 nm. (C) UV/VIS spectra of the compounds.

### *Genome sequencing of Streptomyces sp*. MBK6

**Figure 3.**
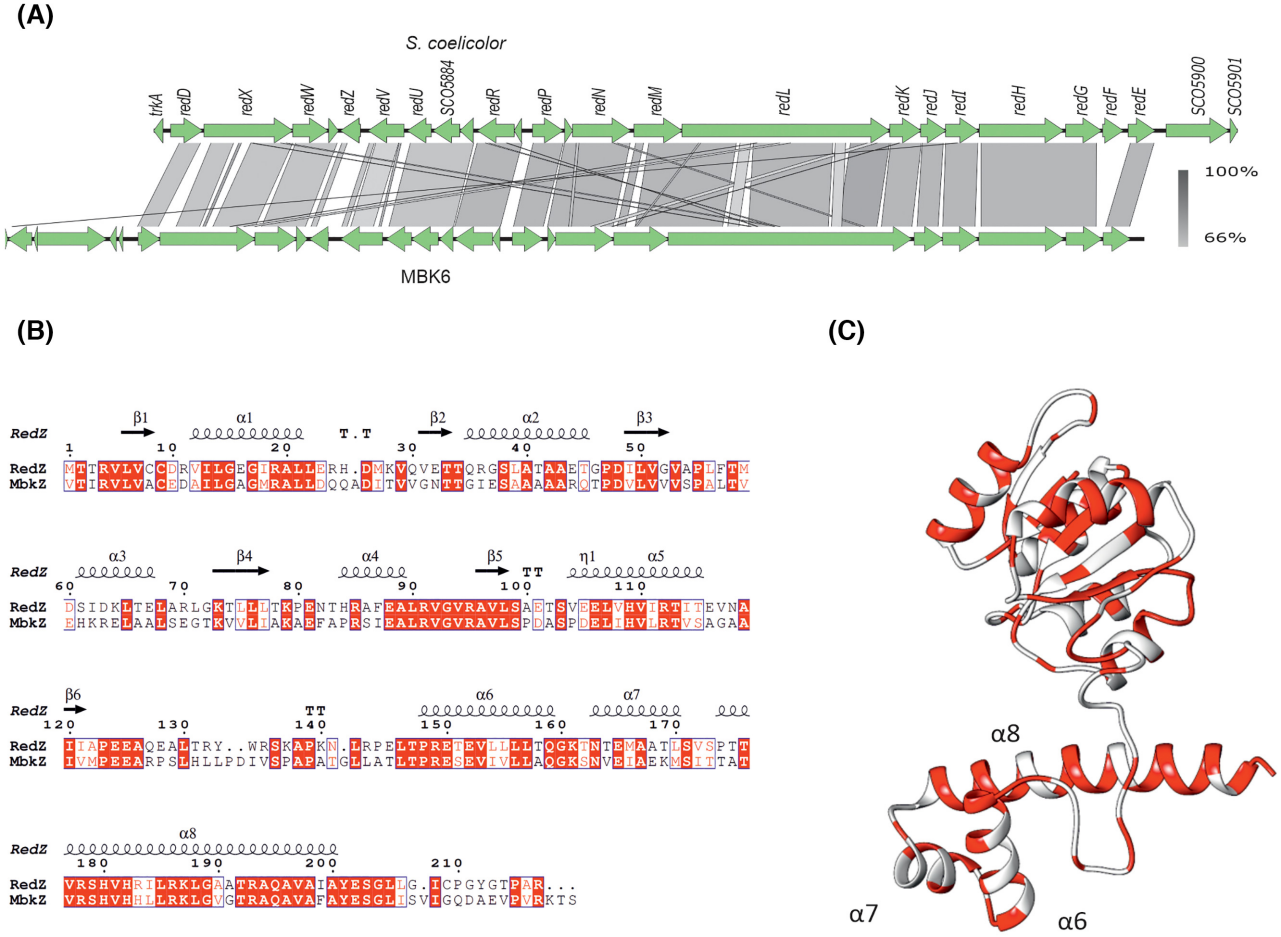
**(A)** EASYFIG (Sullivan *et al*. 2011) comparison of the *S. coelicolor red* cluster and the MBK6 prodigiosin cluster. **(B)** Alignment (Clustal Omega, Sievers and Higgins 2014) of RdmZ and MbkZ annotated with Espript (Robert and Gouet 2014); the secondary structures are derived from the model in panel C. **(C)** Homology model of RedZ, generated by the SwissModel (Waterhouse *et al*. 2018) server using as template 4LDZ (*Bacillus subtilis* response regulator DesR), with amino acids conserved with MbkZ colored red. B.

A draft genome sequence of *Streptomyces* sp. MBK6 acquired using Illumina MiSeq was analysed with antiSMASH (Blin *et al*. 2019). An undecylprodigiosin-type cluster was readily identified (90% of genes showing similarity) among the 26 identified BGCs. The undecylprodigiosin cluster is essentially colinear with the well-characterized *red* cluster of *S. coelicolor* (Malpartida *et al*. 1990; Cerdeño, Bibb and Challis 2001; Hu *et al*. 2016; Fig. 3A). The most significant differences are the lack of a homolog of the oxidoreductase *redF* of unknown function, translational fusion of the type I polyketide synthase *redL* and the oxidoreductase *redK* (Cerdeño, Bibb and Challis 2001) and extension of sequence similarity to SCO5899, which in some publications has been named *redE* (Hu *et al*. 2016). Importantly, we noted that the N-terminal part of *redZ*, the primary regulator of the *red* BGC (Guthrie *et al*. 1998), was not conserved.

A BLAST (Altschul *et al*. 1990) search of the *Streptomyces* clade of the *refseq_genomes* database at NCBI with the *Streptomyces* sp. MBK6 undecylprodigiosin cluster indicated a 98.91% nucleotide sequence identity with a *Streptomyces griseoaurantiacus* strain M045 (Li *et al*. 2011) sequence (NZ_AEYX01000041.1). The 16S rRNA sequence of *Streptomyces* sp. MBK6 has only one base difference from the 16S rRNA of *S. griseoaurantiacus* M045. Comparison of the genomes using Easyfig (Sullivan, Petty and Beatson 2011) revealed extensive colinearity and high sequence similarity, indicating the two strains are closely related (Figure S13, Supporting Information). Production of prodigiosins by *S. griseoaurantiacus* M045 has been observed (Li *et al*. 2005). *S. griseoaurantiacus* M045 has been isolated from marine sediment in China, whereas *Streptomyces* sp. MBK6 has been isolated from a soil sample in Finland.

### Cross-regulation of *S. coelicolor* prodigiosin production by the putative regulator mbkZ

RedZ of *S. coelicolor* is a member of the ‘orphan response regulator’ family (Guthrie *et al*. 1998; Liu *et al*. 2013), in which the conserved phosphorylation site and the cognate histidine kinase gene are missing, and it is the master regulatory protein of the *red* cluster. Thus, the non-conservation of the nucleotide sequence with *mbkZ* suggested different regulation of the prodigiosin production between the strains. On the amino acid level (Fig. 3B and C), the highest similarity is within the DNA-binding domain (Liu *et al*. 2013), whereas the receiver domain shows weaker similarity.

**Figure 4.**
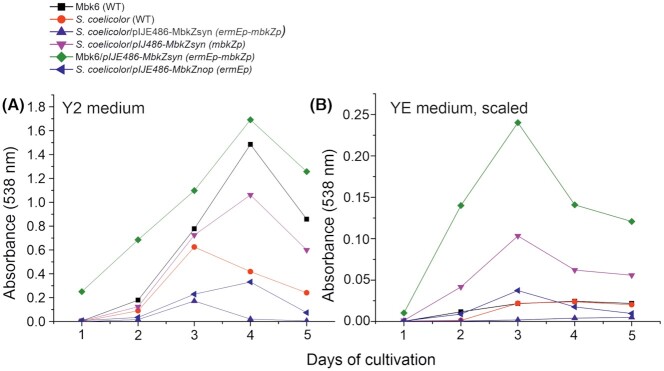
Comparison of prodigiosin production (measured as A_538_ of extracts) of wild-type *S.coelicolor* and *Streptomyces* sp. MBK6 as well as the same transformed with indicated *mbkZ* containing constructs in Y2 medium (with yeast, **A)** and in YE medium (without yeast, **B)**. Panel B is shown with magnified *Y-*axis.

To test this, we decided to transfer *mbkZ* with its own and/or a constitutive promoter to *S. coelicolor* M145. The synthetic gene with promoter was subcloned in pIJE486 (Ylihonko *et al*. 1996; *ermEp-mbkZp*), in pIJ486 (Ward *et al*. 1986; *mbkZp*) and in pIJE486 without its own promoter (*ermEp*) in *S. lividans* TK24 as an intermediate host. Once the plasmids were transformed to *S. coelicolor* M145, the strains were grown in parallel cultures in Y2 medium (containing 2.5 g/L fresh, autoclaved yeast and 2.5 g/L yeast extract) and YE medium (containing 2.5 g/L yeast extract), together with wild-type *S. coelicolor* M145 and *Streptomyces* sp. MBK6. Total prodigiosin production was assayed by absorbance at 538 nm in toluene after alkaline extraction to remove actinorhodin and acidification (adapted from Kim *et al*. (2007)). The constructs were also transformed into *Streptomyces* sp. MBK6 itself, but only for pIJE486-MbkZsyn *(ermEp-mbkZp)* was the transformation successful, despite several attempts. Production of prodigiosins by the various strains in both media is shown in Fig. 4. Wild-type *S. coelicolor* produced 30 times, and *Streptomyces* sp. MBK6 75 times more prodigiosins in Y2 medium than in YE medium. Introduction of pIJ486-MbkZsyn *(mbkZp)* in *S. coelicolor* stimulated production in both Y2 and YE. The construct pIJE486-MbkZsyn *(ermEp-mbkZp)*, containing the strong constitutional, wild-type *ermE* promoter preceding the *mbkZ* promoter region strongly suppressed the production level both in Y2 and YE in *S. coelicolor*, whereas in *Streptomyces* sp. MBK6 it stimulated production in both media. The repression was observed in *S. coelicolor* also with the construct containing only the *ermEp* promoter [pIJE486-MbkZnop *(ermEp)*].

**Figure 5.**
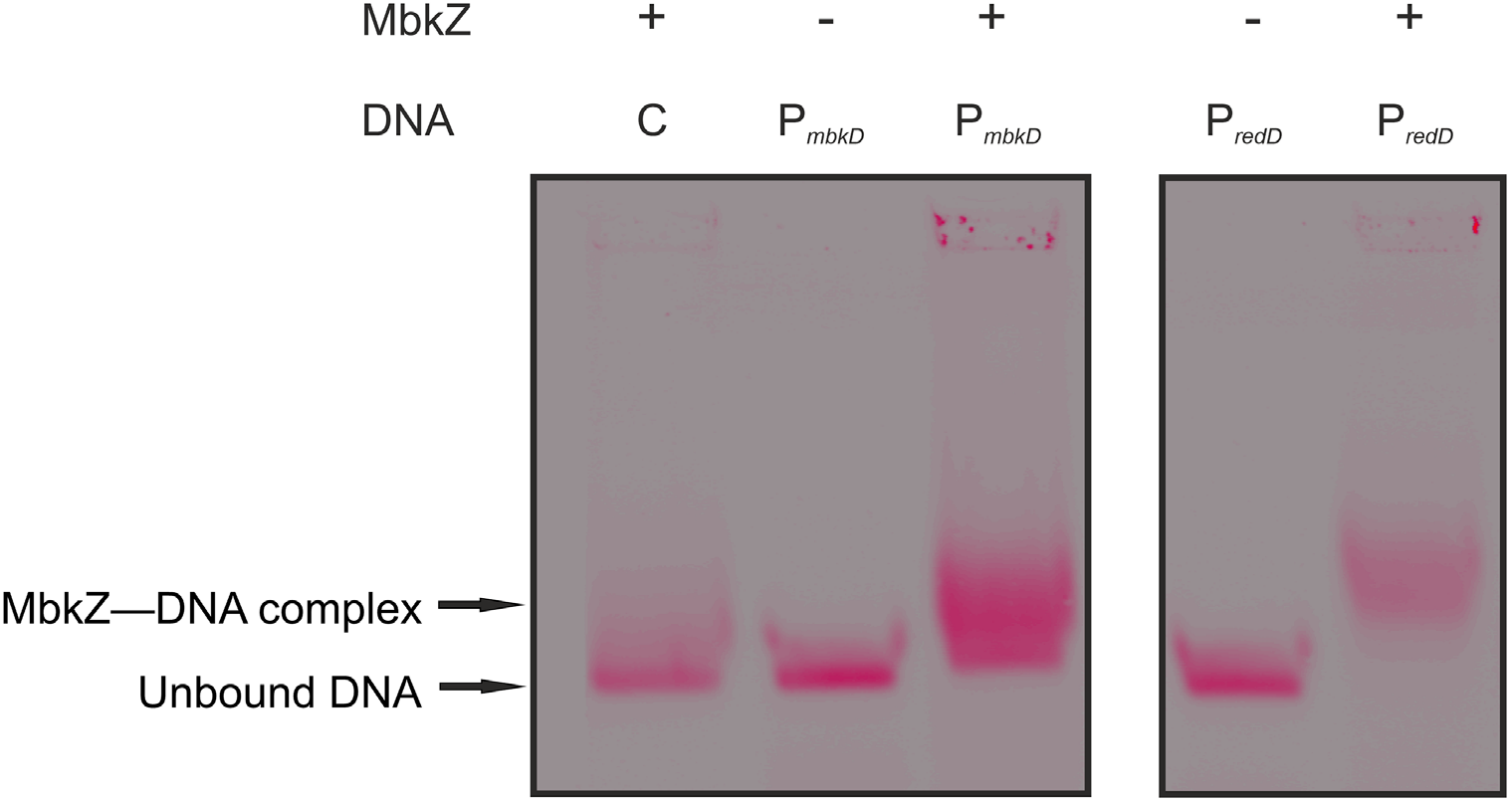
Electrophoretic mobile shift assay (EMSA) of MbkZ protein binding to *mbkD* and *redD* promoters (*mbkDp* and *redDp*, respectively).

The visible effect on the production of prodigiosins of *mbkZ* led us to investigate the binding of MbkZ to the promoter regions of known and putative, respectively, *Streptomyces* antibiotic regulatory proteins (SARP) *redD* and *mbkD* by electrophoretic mobility shift assay (EMSA). Histidine tagged recombinant MbkZ produced in Escherichia coli and purified to near homogeneity by affinity chromatography binds to the promoter region of mbkD as well as to the promoter region of S. coelicolor redD (Fig. 5).

## DISCUSSION

*Streptomycetes* possess a great potential to produce new bioactive substances from latent BGCs, which typically outnumber 10-fold the clusters for observed products. Finding the conditions and signals, which trigger the production of these normally unseen products, is a central research problem. This study originated from experiments attempting to use yeast cells as a stimulus for production. Wild isolates from soil samples were cultured in a medium containing yeast extract as a nitrogen source with or without the supplement of whole autoclaved yeast. The strain designated *Streptomyces* sp. MBK6 was found to produce a red substance in yeast-containing medium. It has subsequently been observed, that at high cell density the pigment is produced also without yeast, but as can be seen in Fig. 4, the production in Y2 medium is around 75 times that observed in YE medium.

The genome sequence of MBK6 confirmed the presence of a prodigiosin BGC, and further revealed that the genome shows a high similarity to a *Streptomyces* strain, *S. griseoaurantiacus* M045 (Li *et al*. 2011) isolated from marine sediment in China. Therefore, *Streptomyces* sp. MBK6 should be classified as a member of the species *S. griseoaurantiacus*. The similarity emphasizes the wide geographical and ecological distribution of *Streptomyces*; search of new isolates has concentrated on exotic habitats, but an essentially identical strain could be isolated from a stereotypical source, ordinary soil.

The two major pigmented products of *Streptomyces* sp. MBK6 were identified as undecylprodigiosin and metacycloprodigiosin. In *S. coelicolor* the cyclized prodigiosin is butylcycloheptylprodigidine (Streptorubin B; Tsao *et al*. 1985). Reaction specificity difference at MbkG, the homolog of RedG, a non-haem iron dependent dioxygenase (Sydor *et al*. 2011), may cause the structural difference. It is notable, that MbkG is more like the metacycloprodigiosin-producing McpG from *S. longispororuber* (Sydor *et al*. 2011) than RedG (Figure S14, Supporting Information). Generally, the *Streptomyces* sp. MBK6 prodigiosin BGC is almost colinear with that of *S. coelicolor*, except that the gene homologous to *redF* is absent from both the gene cluster and the genome. This suggests that the gene is not essential, whereas SCO5899 (*redE*) may have a role in the biosynthesis, as it is conserved in the gene cluster. However, it appears to be an acyltransferase family protein; it has been suggested (Cerdeño, Bibb and Challis 2001) that *redI* is the *O*-methyltransferase that complements the *redE* mutation. Therefore, the function of SCO5899 remains unclear. In *Streptomyces* sp. MBK6, *redL* and *redK* homologs are part of the same ORF.

The most striking difference is the lack of similarity in the N-terminal part of *redZ* and its counterpart *mbkZ*. Prodigiosin biosynthesis in *S. coelicolor* is regulated in many ways at a global level (Liu *et al*. 2013). The transcription of the cluster situated *redZ* is prevented during primary metabolism by two-component sensor kinases systems that control cell division (Som *et al*. 2017) and antibiotic biosynthesis (Sheeler, MacMillan and Nodwell 2005; Lewis *et al*. 2019), such as *mtrA*/*mtrB* and *absA1*/*absA2*, respectively. In addition, the presence of nutrients such as N-acetylglucosamine derived from chitin prevents the activation of *redZ* through the action of DasR (Rigali *et al*. 2008). The interesting feature of prodigiosins is that these cytotoxic metabolites are found intracellularly in *S. coelicolor*, which have led to the proposal that these compounds act to induce controlled cell death in *Streptomyces* (Rigali *et al*. 2008; Tenconi *et al*. 2018). The hypothesis is that upon nutrient depletion certain parts of the mycelium are sacrificed to ensure development of aerial hyphae and spores that guarantee the survival of the colony. Under these circumstances our observation that the presence of yeast induces production of prodigiosins also in *S. coelicolor* is noteworthy.

The situation seems to be different in *Streptomyces* sp. MBK6. This strain appeared to respond to the presence of yeast and sequester yeast cells to the mycelium, which *S. coelicolor* did not do. Therefore, it may be that this physical interaction leads to use of prodigiosins as antifungal agents, which is an activity that has been demonstrated (Stankovic *et al*. 2014).

**Figure 6.**
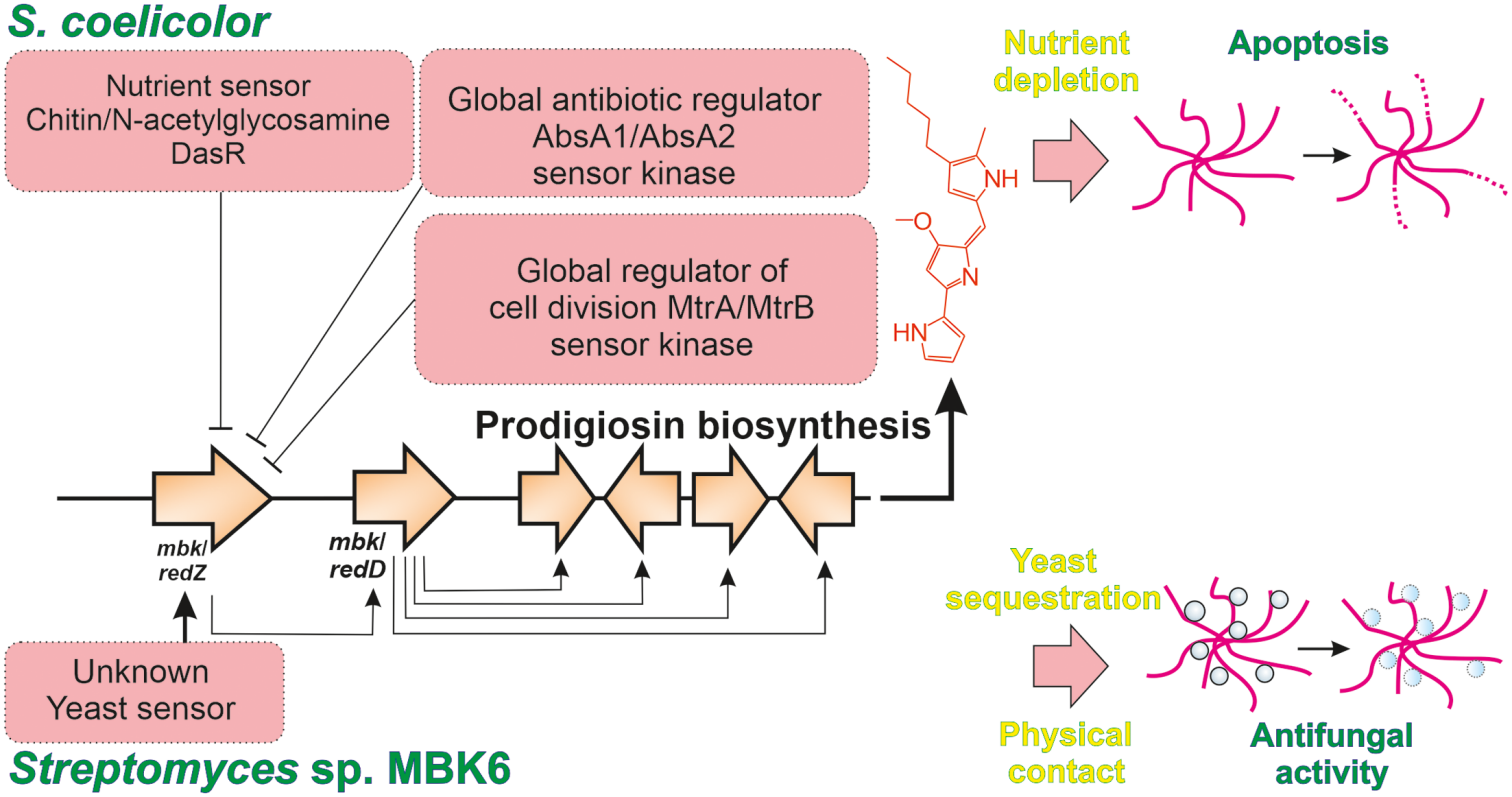
Comparison of postulated regulation of prodigiosin biosynthesis in *S. coelicolor* and *Streptomyces* sp. MBK6

These possible fundamental differences (Fig. 6) may also be reflected on the results from the expression studies and cross-regulation attempts that are presented here. In *Streptomyces* sp. MBK6/pIJE486-MbkZsyn (*ermEp-mbkZp*) constitutive expression of *mbkZ* initiates production of prodigiosins already during day 1 and promotes biosynthesis even in the absence of intact yeast cells. This indicates that the cluster-situated regulatory network is likely to be similar in *Streptomyces* sp. MBK6 and *S. coelicolor*, where MkbZ binds to the promoter region of *mbkD*, which in turn activates the biosynthetic genes. However, the situation seems to be more complex on a global level as revealed by the expression trials of *mbkZ* in *S. coelicolor*. Early expression of *mbkZ* from a constitutive promoter [either *S. coelicolor*/pIJE486-MbkZsyn(*ermEp-mbkZp*) or *S. coelicolor*/pIJE486-MbkZnop(*ermEp*)] seems to lead to repression of prodigiosin production. This may be due to binding of MbkZ to the *redDp* region, which we could observe to occur experimentally, but the inability of MbkZ to function with the *S. coelicolor* RNAP and other transcription factors may prevent activation of prodigiosin production. In addition, MbkZ may also bind to other regions in the genome of *S. coelicolor* that may influence production of antibiotics in unexpected ways. In contrast, expression of *mbkZ* from its natural promoter in *S. coelicolor*/pIJ486-MbkZsyn(*mbkZp*) enhances production of prodigiosins. We speculate that this may be due to a gene dose effect and particularly the presence of multiple *mbkZp* promoter sequences in the multi-copy number plasmid (Kieser *et al*. 1982). These *trans* acting elements may bind the natural repressors of the prodigiosin pathway such as MtrB, AbsA2 and DasR, which would allow production of RedZ and activation of *redD* transcription.

In conclusion, the work provides an intriguing account on the prevalence and diversification of soil microbes. On the one hand, we show the isolation of near identical *Streptomyces* strains from vastly different geographical locations and habitats, and the presence of a prodigiosin gene cluster that is highly similar to the one residing in the model organism *S. coelicolor*. However, changes in the receiver domain of the key regulatory protein MbkZ has led to adaptation and differential regulation of production of prodigiosins in response to varied environmental signals.

## Supplementary Material

fnab044_Supplemental_File
