# Supplementary material for: Differential regulation of undecylprodigiosin biosynthesis in the yeast-scavenging Streptomyces strain MBK6
Source: FEMS Microbiol Lett. 2021 Apr 21;368(8):fnab044. doi: 10.1093/femsle/fnab044 (PMC8102152; doi:10.1093/femsle/fnab044)
Supplement: fnab044_Supplemental_File [file fnab044_supplemental_file.docx]

# Supporting information

Differential regulation of undecylprodigiosin biosynthesis in the yeast-scavenging *Streptomyces* strain MBK6

Baral, B., Siitonen, V., Laughlin, M., Yamada, K., Ilomäki, M., Metsä-Ketelä, M., Niemi, J.

*Table of Contents*

Supplementary experimental procedures

Table S1. NMR-data for metacycloprodigiosin (**1**) and undecylprodigiosin (2). Figure S1. Main HMBC and COSY correlations for **1** and **2**.

Figure S2. Proton spectrum of **1** in MeOD.

Figure S3. Carbon spectrum of **1** in MeOD.

Figure S4. COSY spectrum of **1** in MeOD.

Figure S5. HSQCDE spectrum of **1** in MeOD.

Figure S6. HMBC spectrum of **1** in MeOD.

Figure S7. Proton spectrum of **2** in CDCl_3_

Figure S8. Carbon spectrum of **2** in CDCl_3_

Figure S9. COSY spectrum of **2** in CDCl_3_.

Figure S10. HSQCDE spectrum of **2** in CDCl_3_.

Figure S11. HMBC spectrum of **2** in CDCl_3_.

Figure S12. HR-MS spectra of **1** and **2**.

Figure S13. Alignment of selected contigs from MBK6 and M045

Figure S14. Alignment and similarity tree of RedG homologs.

References

### Synthesized genes

The *mbkZ* ORF with its promoter (1057 bp) cloned in pUC57 plasmid (pUC57MbkZ) was purchased in string form from Genewiz (South Plainfield, NJ 07080, USA). The native sequence was modified (indicated in green) to introduce a *Bam*HI site (TT -> CC), *Kpn*I site (TCCCA -> GTACC) and a *Hin*dIII site (added AAGCTTCAT)

1 ATCGGATCCCCGGGCCGCGAACACCGGCCATTCCACGTACTTCGACAATCCGCGGTGGCTCGATTTCGCTCCCCCGTGAA 80

BamHI

81 CGAAGGCGACCTATACGGAATATTGCGTGGGTCACAGACGACTGACACAATCAGTCGCCCCAGGCAGCAAGGAGTGTCGG 160

161 AAGAGCGCTGCGGCGCCCTCTCGGGGGAGGCACGTACTTTGCAGGACCGCAAGCCGTCACAACCGGAAAAGACCGCCTGC 240

241 CGGTTCACCTGGTCGGTAAAACCTGCCATCGCCCGATGAGGGCATCTGCGACCAACGCACACTCCGGGCCGAGCCGGGTC 320

V T I R V L V A C E D A

321 GACCACCCTTGCACCCACTCGGGTACCCCTCACGGGAGGCAGTCGGTGACAATTCGCGTACTCGTGGCTTGCGAGGATGC 400

KpnI

I L G A G M R A L L D Q Q A D I T V V G N T T G I E

401 GATCCTGGGCGCCGGGATGCGCGCGCTGCTCGACCAGCAGGCCGACATCACCGTGGTCGGCAACACGACCGGCATCGAGT 480

S A A A A A R Q T P D V L V V V S P A L T V E H K R E

481 CCGCGGCGGCCGCCGCGCGGCAGACCCCCGACGTGCTCGTGGTCGTCTCCCCCGCGCTGACCGTGGAGCACAAACGGGAG 560

L A A L S E G T K V V L I A K A E F A P R S I E A L R

561 CTCGCCGCGCTGTCGGAGGGCACGAAGGTCGTGCTCATCGCGAAGGCCGAGTTCGCGCCCCGCTCCATCGAGGCCCTGCG 640

V G V R A V L S P D A S P D E L I H V L R T V S A G

641 GGTCGGCGTCAGGGCGGTGCTGAGTCCGGACGCCTCGCCCGACGAGCTGATCCACGTCCTGCGGACGGTCAGCGCCGGCG 720

A A I V M P E E A R P S L H L L P D I V S P A P A T G

721 CGGCCATCGTCATGCCGGAGGAGGCCCGGCCGAGCCTGCACCTCCTCCCGGACATCGTCTCCCCCGCGCCCGCCACGGGA 800

L L A T L T P R E S E V I V L L A Q G K S N V E I A E

801 CTGCTGGCCACGCTGACCCCCCGGGAATCCGAGGTCATTGTGCTGCTCGCCCAGGGGAAGTCCAATGTCGAGATAGCGGA 880

K M S I T T A T V R S H V H H L L R K L G V G T R A

881 GAAGATGTCCATAACCACGGCGACGGTACGGTCCCACGTTCACCATCTGCTGAGAAAGCTCGGGGTGGGGACGCGCGCCC 960

Q A V A F A Y E S G L I S V I G Q D A E V P V R K T S

961 AGGCGGTTGCCTTCGCCTACGAGTCGGGCCTCATTTCCGTCATTGGACAGGACGCGGAGGTCCCGGTGCGGAAAACCTCC 1040

*

1041 TGACGGCCAAGCTTCAT 1057

HindIII

### Plasmid constructions

*pIJE486-MbkZsyn (ermEp-mbkZp) and* *pIJ486-MbkZsyn (mbkZp):*

pUC57MbkZ was digested with *Bam*HI and *Hin*dIII and the smaller fragment ligated with the larger fragment of pIJE486 (Ylihonko *et al.* 1996) or pIJ486 (Ward *et al.* 1986), respectively. The ligations were then transformed into *S. coelicolor* M145 and *S. lividans* TK24 through protoplast transformation (Kieser *et al.* 2000).

*pBADHisBΔMbkZ:*

pBADHisBΔ (Kallio *et al.* 2006) was digested with *Kpn*I and *Hin*dIII, the large fragment isolated, ligated with the smaller fragment from a digestion of pUC57MbkZ with *Kpn*I and *Hin*dIII and transformed into *E. coli* TOP10. The construct was confirmed by restriction with *Kpn*I and *Hin*dIII. This construct encodes a N-terminal his-tag and 12 amino acids before the start of the native peptide sequence:

MAHHHHHHHRSAAGTPHGRQSVTIRVLVACEDAILGAGMRALLDQQADITVVGNTTGIESAAAAA

RQTPDVLVVVSPALTVEHKRELAALSEGTKVVLIAKAEFAPRSIEALRVGVRAVLSPDASPDELI

HVLRTVSAGAAIVMPEEARPSLHLLPDIVSPAPATGLLATLTPRESEVIVLLAQGKSNVEIAEKM

SITTATVRSHVHHLLRKLGVGTRAQAVAFAYESGLISVIGQDAEVPVRKTS

*pIJE486-MbkZnop (ermEp)*

pBADHisBΔMbkZ was digested with *Bgl*II and *Hin*dIII, the smaller fragment was cloned into pIJE486 (*Bam*HI-*Hin*dIII fragment) and transformed as above.

### Primers

REDDPROM1 5’-DY682-GAACCGAGGCGACGGAAGGAGG-3’

REDDPROM2 5’-GGTCCATCGTGGCAAGCACTCCC-3’

MBKDPROM1 5’-DY682- AGATGGCGTCTGGGAACCGCTGG-3’

MBKDPROM2 5’-CACCTTGTTGGCGCGAATGGCGTG-3’

CONMBKX1 5’-DY682- CCGGACAGTTCTGGGAATTGA-3’

CONMBKX2 5’-GCGACGTCGACGGGGAT-3’

Table S1. NMR-data for metacycloprodigiosin (**1**) and undecylprodigiosin (**2**). **1** was recorded in MeOD, proton at 500 MHz carbon at 151 MHz and **2** was recorded in CDCl_3_, proton at 500 MHz carbon at 126 MHZ. The signals are referenced to tetramethylsilane.

| **1** |  |  | **2** |  |  |
| --- | --- | --- | --- | --- | --- |
| Position | δ ppm | δ ppm, *J* Hz |  | δ ppm | δ ppm, *J* Hz |
|  | 13C | ^1^H | Position | 13C | 1H |
| 1 | ND |  | 1 | ND |  |
| 2 | 112.2 | 6.31, br | 2 | 111.9 | 6.37, d 3.8 |
| 3 | 150.1* |  | 3 | 129.4 | 6.16, d 3.8 |
| 4 | 128.8 |  | 4 | ND |  |
| 5 | - |  | 5 | - | 13.8, br |
| 6 | 114.3 | 7.01, s | 6 | 116.2 | 6.95, s |
| 7 | ND |  | 7 | ND |  |
| 8 | 169.2 |  | 8 | 169.5 |  |
| 8-O-CH_3_ | 59.5 | 4.03, s | 8-O-CH_3_ | 58.7 | 4.01, s 3H |
| 9 | 95.1 | 6.35, m | 9 | 93.6 | 6.07, s |
| 10 | 154.6* |  | 10 | ND |  |
| 11 | - |  | 11 | - |  |
| 12 | 126.5* |  | 12 | ND |  |
| 13 | 115.0 | 7.02, m | 13 | 118.1 | 6.92 d, 4.4 |
| 14 | 112.3 | 6.35, m | 14 | 111.9 | 6.36 dd, 4.4, 2.5 |
| 15 | 125.7 | 7.14, m | 15 | 127.5 | 7.29, br |
| 16 | - |  | 16 | - |  |
| 1´ | 30.0 | 2.79, m, 2H | 1´ | 28.6 | 2.77 m, 2H |
| 2´ | 28.1 | 1.62, m; 1.84, m | 2´ | 29.3 | 1.72 m |
| 3´ | 28.9 | 1.24, m; 1.46, m | 3´–8´´ | 29.4–29.9 | 1.25–1.38, m |
| 4´ | 26.8 | 0.24, m; 0.82, m | 9´´ | 32.3 | 1.25 m |
| 5´ | 25.9 | 1.05, m; 1.34, m | 10´´ | 22.9 | 1.28 m |
| 6´ | 23.9 | 0.96, m; 1.38, m | 11´ | 14.1 | 0.88 t 7.1 |
| 7´ | 27.9 | 0.95, m; 1.10, m |  |  |  |
| 8´ | 35.3 | 1.45, m; 1.75, m |  |  |  |
| 9´ | 40.9 | 2.55, m |  |  |  |
| 10´ | 31.1 | 1.62, m; 1.69, m |  |  |  |
| 11´ | 13.0 | 0.88, t, 7.4 |  |  |  |

Integrals are not reliable in the region below 2 due to overlapping signals and trace impurities otherwise integrals are 1H, unless otherwise stated.

m = multiplet, d = doublet, s = singlet, br = broad signal, ND = no data *Values gotten from 2D spectra, not the ^13^C spectrum.

NH

HN

N

O

1

2

H

N

NH

N

O

Figure S1. Main HMBC and COSY correlations for **1** and **2**. HMBC correlations are presented with blue arrows and COSY correlations with pink lines.


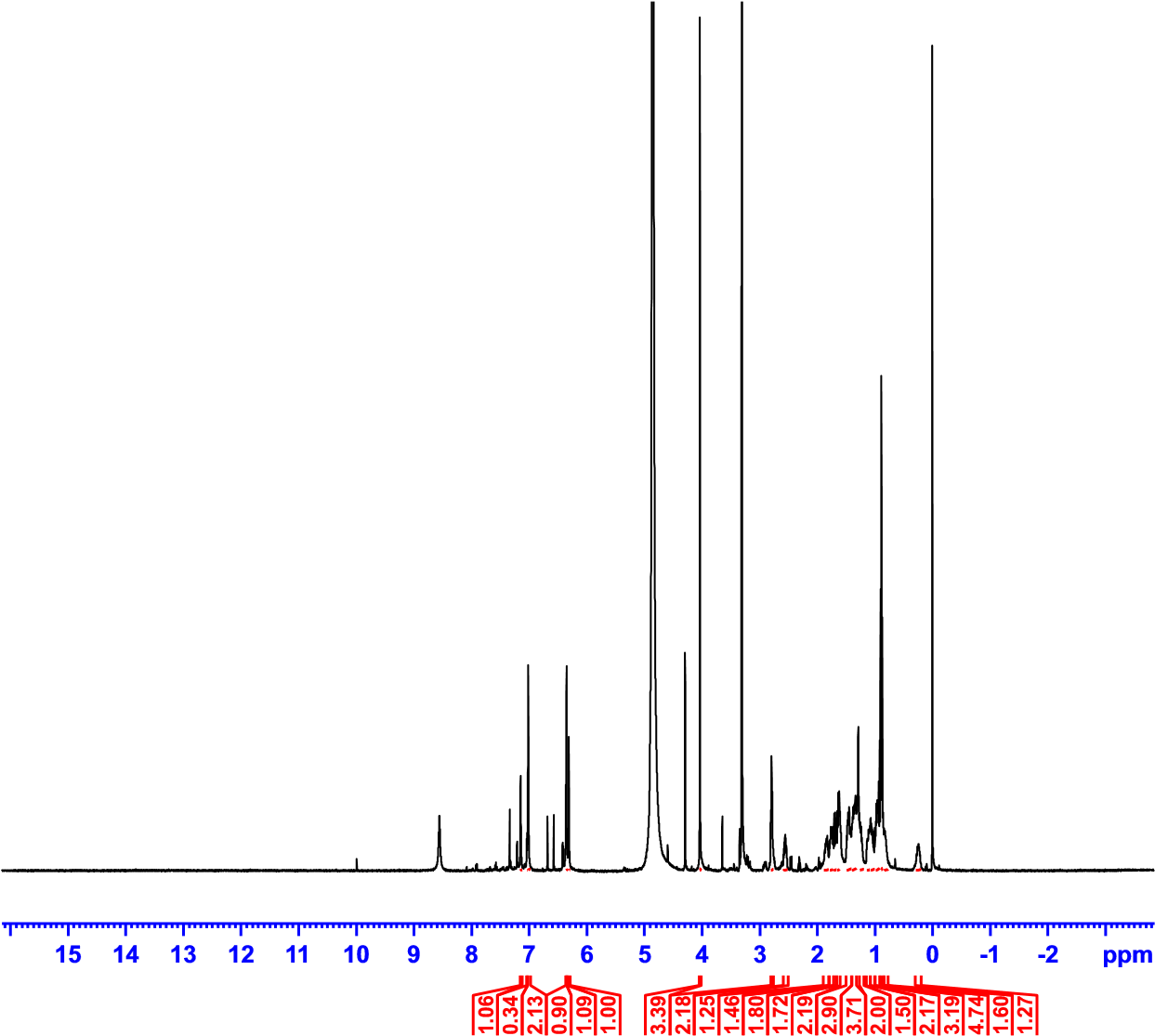


Figure S2. ^1^H spectrum of **1** in MeOD.

**ppm**

**[**

**]**

**150**

**100**

**50**

**0**

**rel**

**]**

**[**

**0.00**

**0.05**

**0.10**

**0.15**

**0.20**

**0.25**

**169.0480**

**128.6833**

**127.1070**

**1**

**14.2571**

**1**

**13.1270**

**1**

**12.3234**

**96.7018**

**95.1093**

**59.4995**

**49.4714**

**49.3315**

**49.1875**

**49.0462**

**48.9061**

**48.7613**

**48.621**

**1**

**40.9085**

**35.4758**

**35.3315**

**31.0616**

**30.9871**

**30.2963**

**29.9838**

**28.7800**

**28.0720**

**27.8277**

**27.7247**

**26.7431**

**25.7891**

**23.8408**

**13.0279**

**0.0000**

Figure S3. ^13^C spectrum of **1** in MeOD.


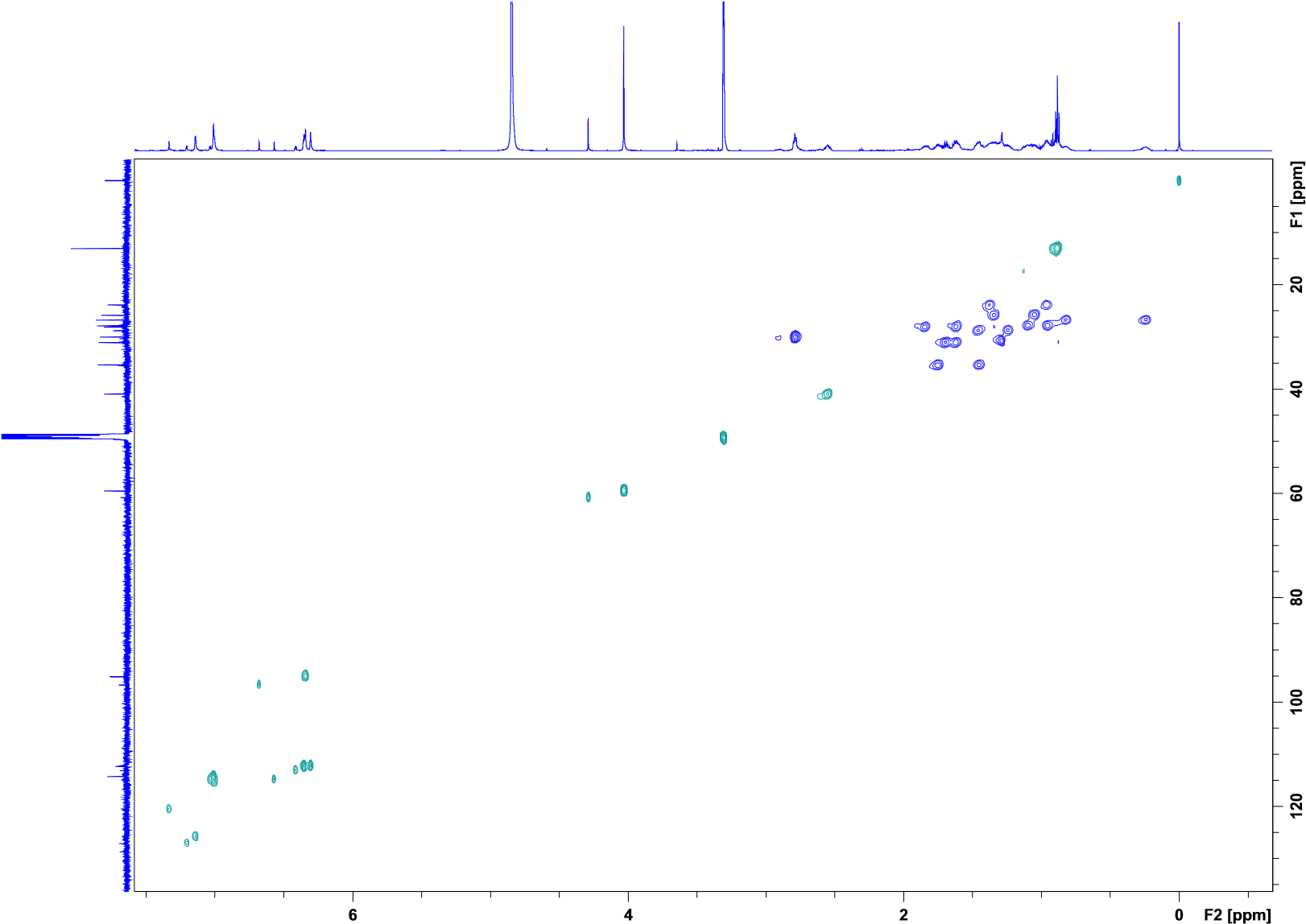
Figure S4. HSQCDE spectrum of **1** in MeOD.


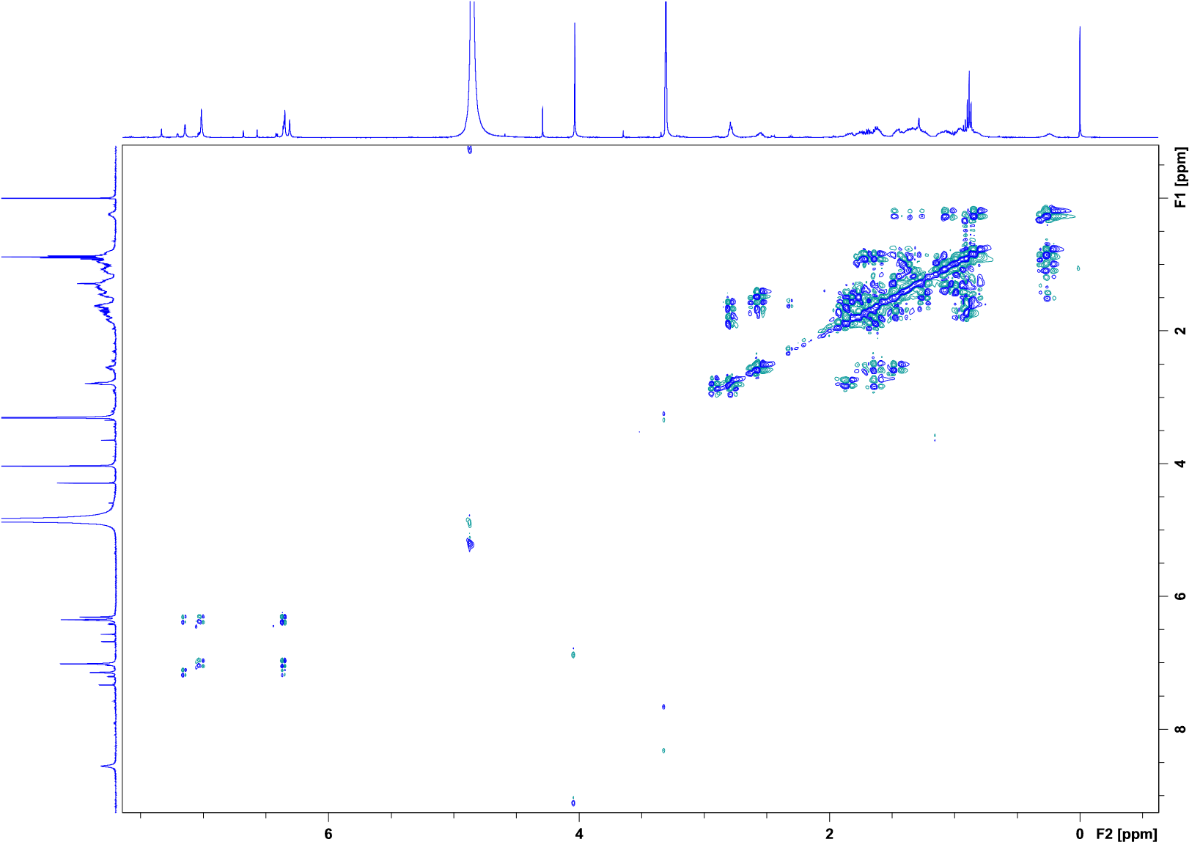


Figure S5. COSY spectrum of **1** in MeOD.


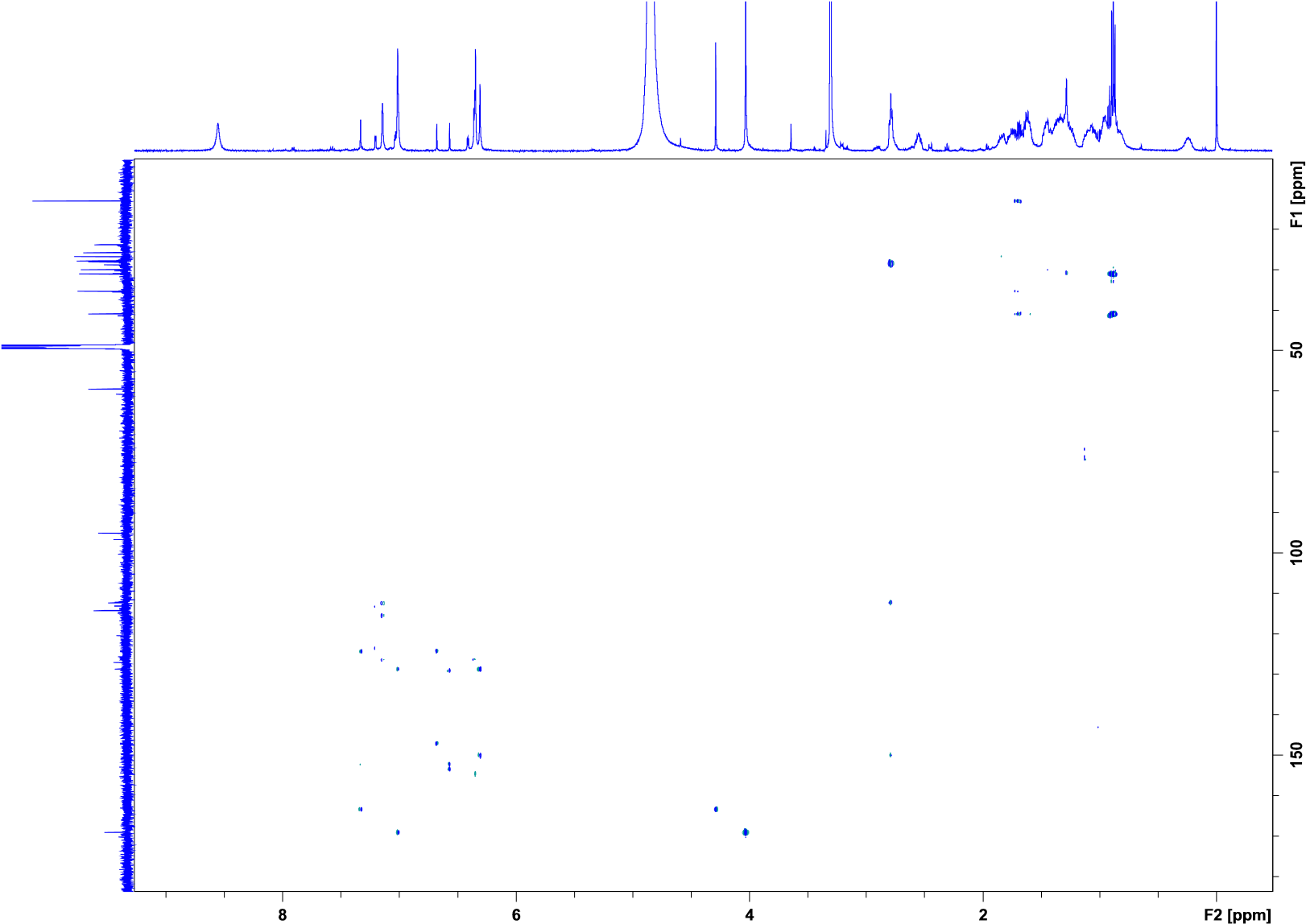
Figure S6. HMBC spectrum of **1** in MeOD.


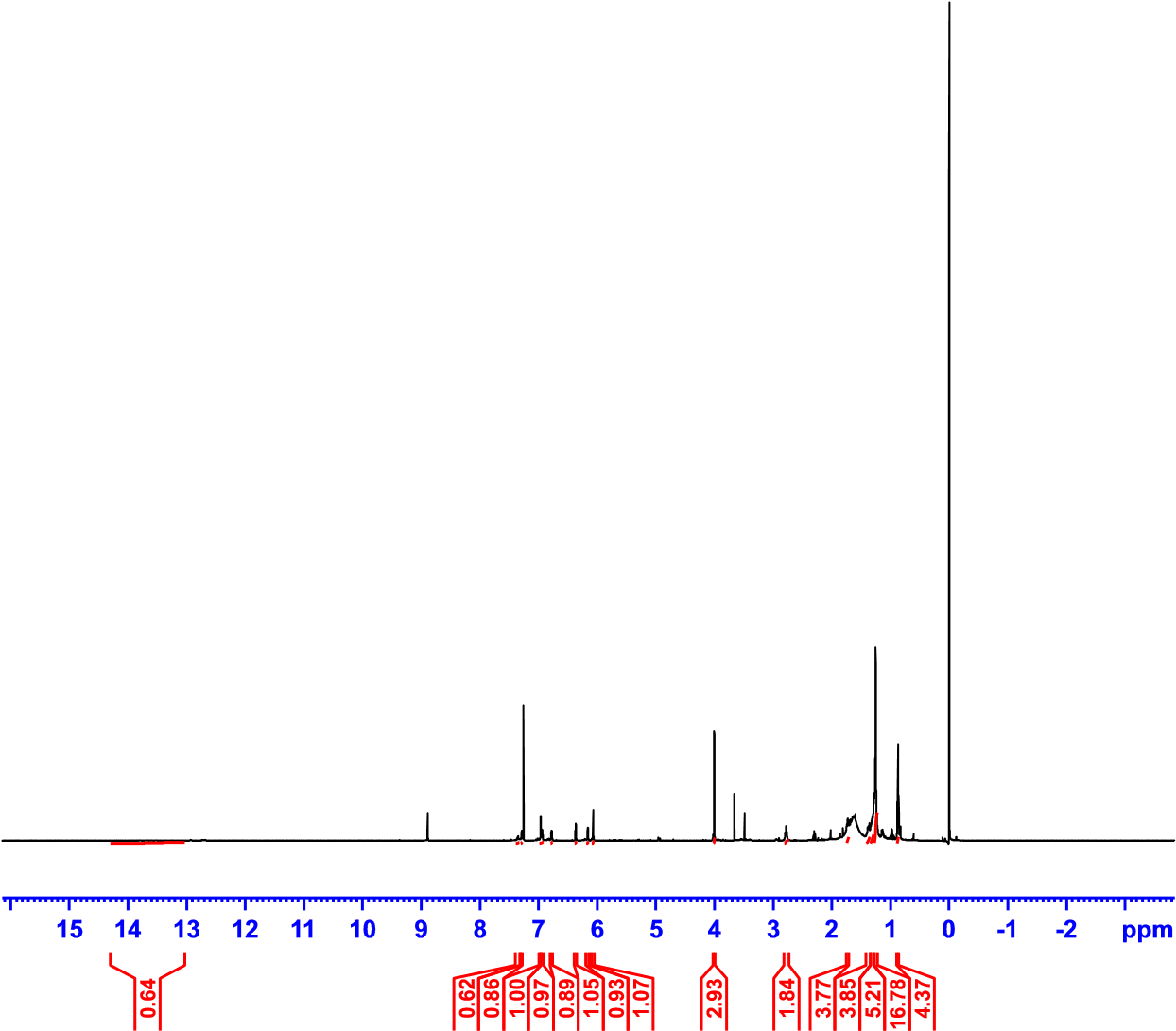


Figure S7. ^1^H spectrum of **2** in CDCl_3_.

Figure S8. ^13^C spectrum of **2** in CDCl_3_.

**]**

**[**

**ppm**

**200**

**150**

**100**

**50**

**0**

**rel**

**[**

**]**

**-**

**0.0**

**0.2**

**0.4**

**0.6**

**169.2992**

**1**

**16.1862**

**1**

**1**

**1.6800**

**93.5845**

**77.2713**

**77.0176**

**76.7638**

**58.6698**

**31.9152**

**29.6389**

**29.6259**

**29.5604**

**29.4448**

**29.41**

**19**

**29.3431**

**29.2943**

**28.6417**

**27.1**

**191**

**22.6923**

**19.2398**

**14.1**

**165**

**1**

**1.4126**

**-0.0033**


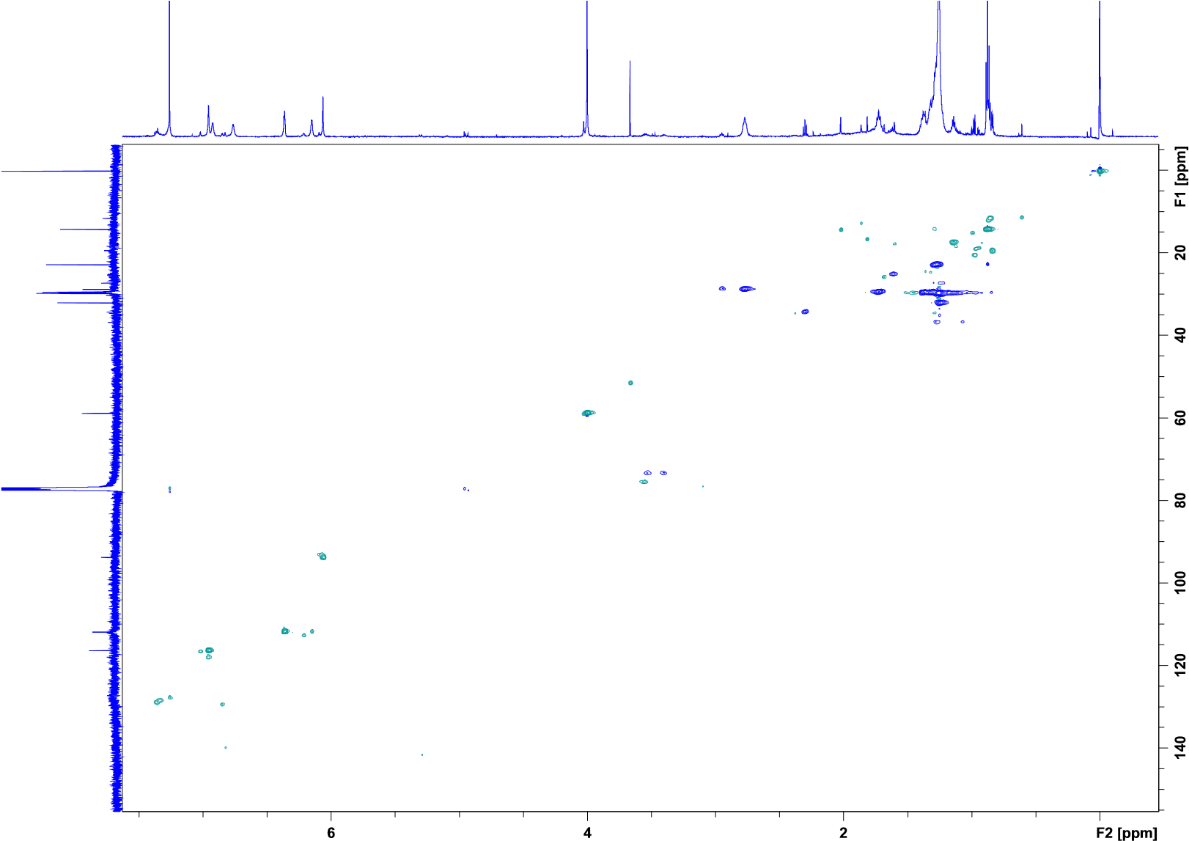


Figure S9. HSQCDE spectrum of **2** in CDCl_3_.


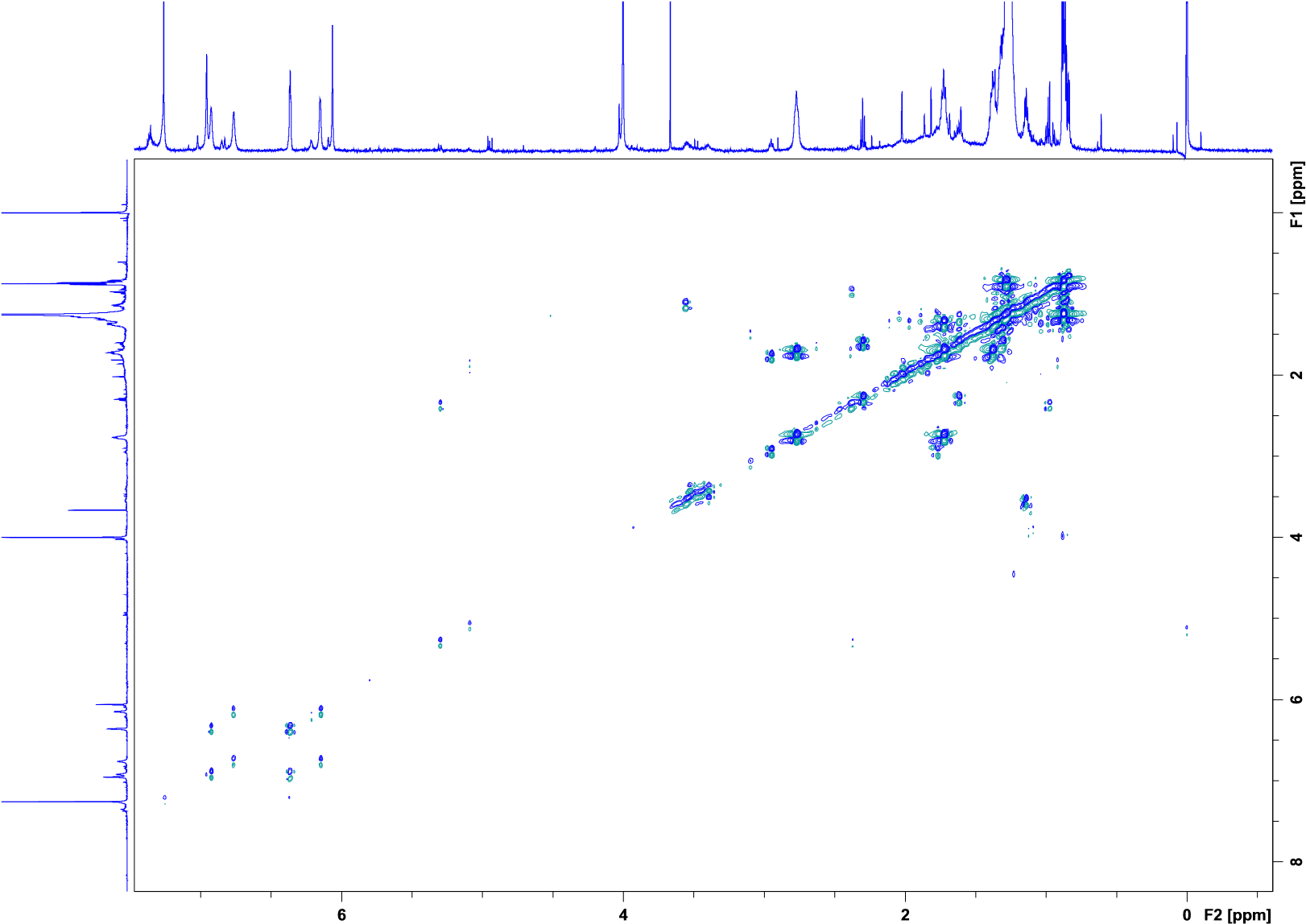
Figure S10. COSY spectrum of **2** in CDCl_3_.


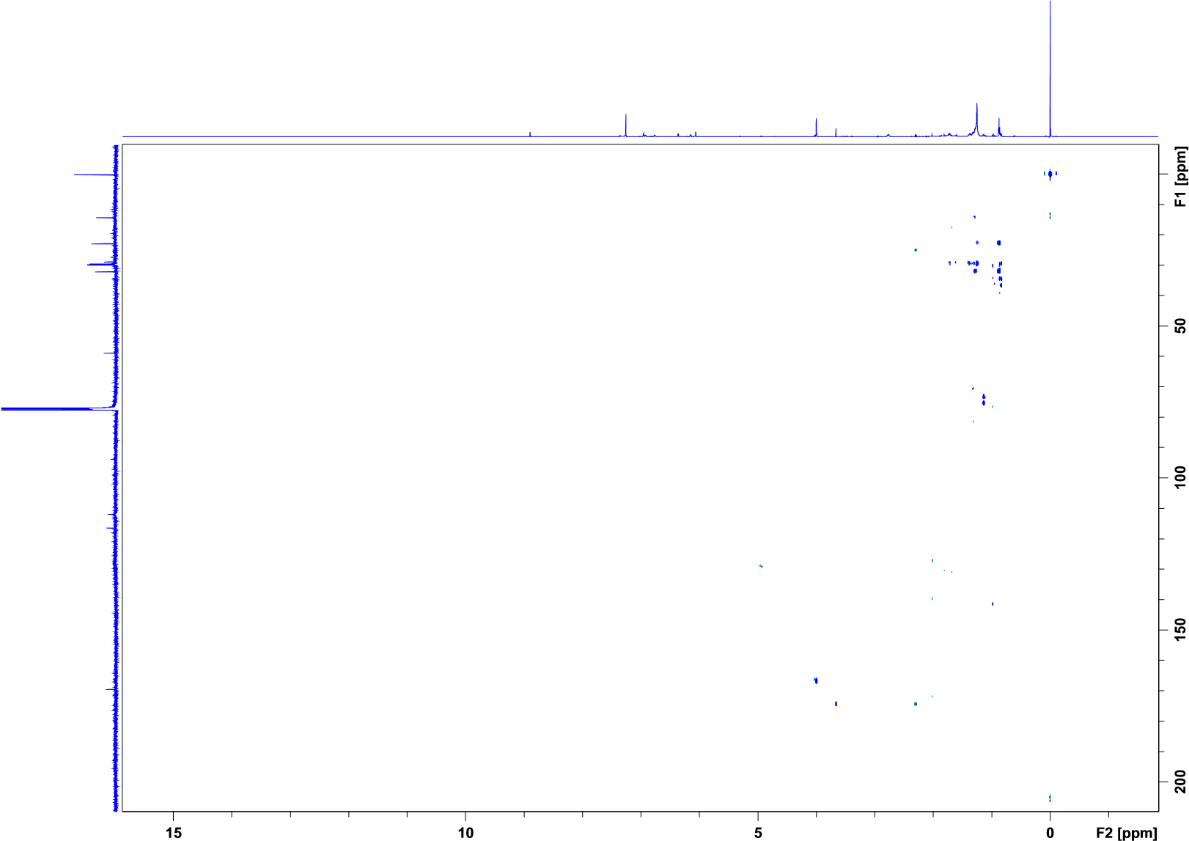


Figure S11. HMBC spectrum of **2** in CDCl_3_.

A


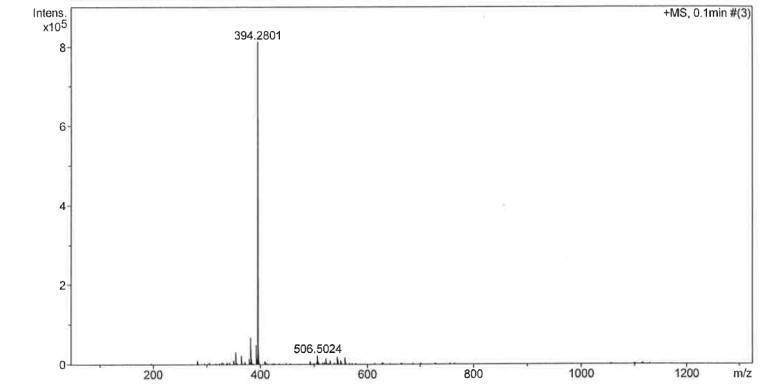

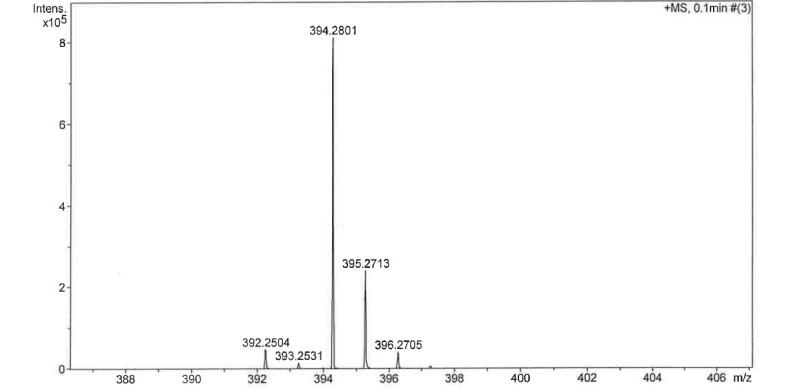


B


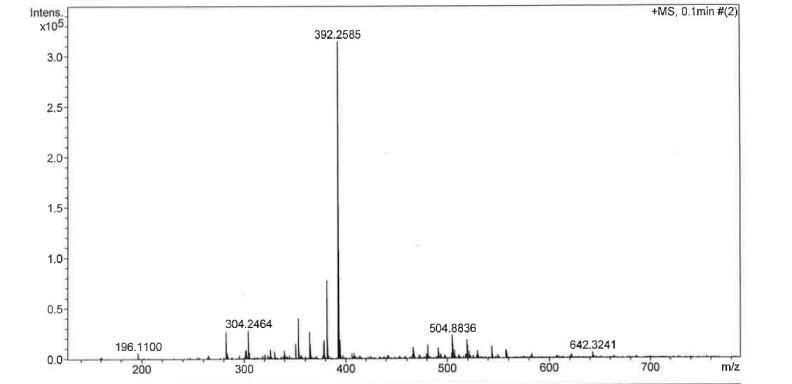

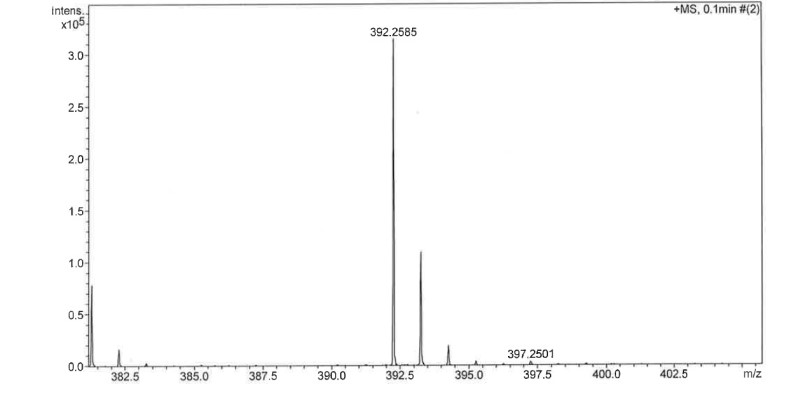


Figure S12. HR-MS spectrum of the **1** and **2**. A) HR-MS for **1**, ESI m/z [M+H]^+^, ESI + obs. 392.2585, calc. 392.2696. B) HR-MS for **2**, ESI m/z [M+H]^+^ , ESI + obs. 394.2801, calc. 394.2853.


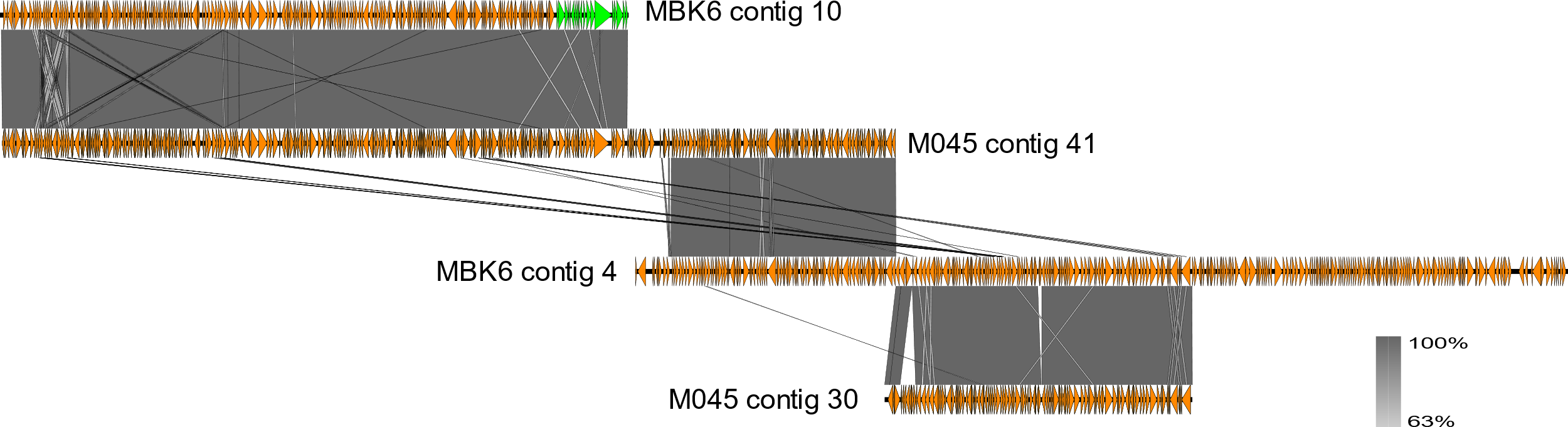


Figure S13. Easyfig (Sullivan *et al.* 2011) alignment of selected contigs from *Streptomyces* sp. MBK6 and *Streptomyces griseoaurantiacus* M045. The prodigiosin BGC in MBK6 is indicated in green.

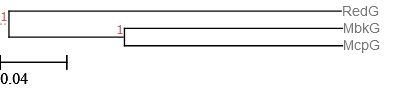


Figure S14. Multiple sequence alignment (Clustal Omega, Sievers and Higgins, 2014) annotated with ESPript (Robert and Gouet, 2014) of the RedG homologs and similarity tree (<http://etetoolkit.org/treeview/>) of the sequences.

References

Kallio, P., Sultana, A., Niemi, J., Mäntsälä, P., and Schneider, G. (2006) Crystal structure of the polyketide cyclase AknH with bound substrate and product analogue: implications for catalytic mechanism and product stereoselectivity*. J Mol Biol* **357:** 210-220.

Kieser, T., Bibb, M., Buttner, M., Chater, K., and Hopwood, D.A. (2000) *Practical Streptomyces genetics*. Norwich, UK: The John Innes Foundation.

Robert, X., and Gouet, P. (2014) Deciphering key features in protein structures with the new ENDscript server*. Nucleic Acids Res* **42:** W320-W324.

Sievers, F., and Higgins, D.G. (2014) Clustal omega*. Curr Protoc Bioinformatics* **48:** 3.13.1-16.

Sullivan, M.J., Petty, N.K., and Beatson, S.A. (2011) Easyfig: a genome comparison visualizer*. Bioinformatics* **27:** 1009-1010.

Ward, J.M., Janssen, G.R., Kieser, T., Bibb, M.J., Buttner, M.J., and Bibb, M.J. (1986) Construction and characterisation of a series of multi-copy promoter-probe plasmid vectors for Streptomyces using the aminoglycoside phosphotransferase gene from Tn5 as indicator*. Mol Gen Genet* **203:** 468-478.

Ylihonko, K., Tuikkanen, J., Jussila, S., Cong, L., and Mäntsälä, P. (1996) A gene cluster involved in nogalamycin biosynthesis from Streptomyces nogalater: sequence analysis and complementation of early-block mutations in the anthracycline pathway*. Mol Gen Genet* **251:** 113-120.
